# Cyclodextrin-Based Polymer-Supported Bacterium for the Adsorption and *in-situ* Biodegradation of Phenolic Compounds

**DOI:** 10.3389/fchem.2018.00403

**Published:** 2018-09-11

**Authors:** Abdalla H. Karoyo, Jian Yang, Lee D. Wilson

**Affiliations:** ^1^Department of Chemistry, University of Saskatchewan, Saskatoon, SK, Canada; ^2^Drug Discovery and Development Research Group, College of Pharmacy and Nutrition, University of Saskatchewan, Saskatoon, SK, Canada

**Keywords:** β-cyclodextrin, polymer, phenolic compounds, adsorption, biodegradation, bacterium immobilization

## Abstract

Dual function polymer materials with immobilized *Sphingobium Chorophenolicum* (SpC) bacterium cells are reported herein that undergo tandem adsorption and biodegradation of phenolic compounds. The cross-linked polymer materials contain β-cyclodextrin (β-CD) with incremental hexamethylene diisocyanate (HDI) cross-linker at variable mole ratios (X = 1, 3, or 6), denoted as HDI-X systems. The adsorptive uptake properties of the insoluble HDI-X polymers (X = 3 and 6) with various phenolic compounds [pentachlorophenol (PCP), 2,4,6-trichlorophenol (TCP), and 2,4,6-trimethylphenol (TMP)] were studied using batch adsorption isotherms. The molecular selective phenol removal (S_R_) capacity of the HDI-3 and HDI-6 materials was evaluated by electrospray ionization mass spectrometry (ESI-MS). The results were compared against granular activated carbon (GAC) and native β-CD, where 1D/2D ^1^H NMR spectral characterization of the complexes formed between phenolic guests and a soluble polymer (HDI-1) in aqueous solution provide insight on the intermolecular interactions and the role of cross-linking effects. Immobilization of SpC onto HDI-3 was shown to form a composite polymer/bacterium material. The composite system displays synergistic removal effects due to tandem PCP adsorption and SpC biodegradation to yield by-products such as 2,6-dichloro-1,4-hydroquinone (DCHQ). Apoptosis and cytotoxicity of DCHQ were evaluated using three breast cancer cell lines.

## Introduction

Chlorophenols and chloroarenes have been widely used as low-cost and effective pesticides in agriculture and the timber industry over the last century (Dorsey and Tchounwou, 2004). In particular, the production of pentachlorophenol (PCP) stands at ca. 50, 000 metric tons yearly despite its ban in the 70's by Japan and Sweden, as a consequence of fish contamination and other related health concerns (Geyer et al., 1986). A subsequent ban by the USA Environmental Protection Agency (US-EPA) in 1987 resulted due to toxicity concerns to fish, livestock, and humans (Wild et al., 1993; Dorsey and Tchounwou, 2004). The high degree of chlorination of PCP accounts for its recalcitrance and resistance to biodegradation, as evidenced by trace levels of PCP detected in soil, water, air, and food (Hattemer-Frey and Travis, 1989). Exposure of PCP to humans often occurs via the skin, respiratory and gastrointestinal tracts, where the health effects of PCP relate to its long half-life (ca. 2–16 days) in human receptors (Reigner et al., 1992). Extended exposure is associated with cancers, miscarriages in women, and immunal, neurological, and endocrine disorders (Zheng et al., 2015). The carcinogenicity of PCP is attributed to the formation of key reactive metabolites such as quinone, hydroquinone and semi-quinone compounds.

Physical and chemical remediation methods such as adsorption by porous carbonaceous solids (Liu et al., 2004; Abdel Salam and Burk, 2010), photocatalytic degradation (Hanna et al., 2004), and liquid-liquid extraction methods (Khodadoust et al., 1999) have been adopted to remediate environmental PCP contamination. However, these methods are limited by scalability, operational and regeneration costs, and possible introduction of toxic secondary by-products. By contrast, biological treatment of soil and water using injectable/infusible bacterial microorganisms offers a clean and sustainable bioremediation strategy for PCP (Banat, 1995). The biodegradation of chlorophenols by various microorganisms such as *Pseudomonas* sp., *Sphingomonas* sp., *Alcaligenes* sp., and *Sphingobium Chlorophenolicum* have been reported (Olaniran and Igbinosa, 2011; Arora and Bae, 2014; Lopez-Echartea et al., 2016). In particular, the mechanism and metabolic pathway for the biodegradation of PCP and its derivatives using *Sphingobium Chlorophenolicum* (SpC) have also been studied (Copley et al., 2012; Arora and Bae, 2014). Despite the advantages associated with biological treatment, the technology is often limited by factors that attenuate the catalytic efficiency. However, the use of synthetically engineered polymer adsorbents, in conjunction with microbes, offers a practical method for the removal of waterborne chlorophenols and their reactive metabolites from the environment. Cyclodextrin (CD) polymer adsorbents are versatile because they offer unique binding properties that can facilitate the design of composite materials essential for the adsorption and *in-situ* biodegradation of phenolic contaminants. CDs are cyclic oligosaccharides with α-1,4 linkages that contain 6 (α-CD), 7 (β-CD), and 8 (γ-CD) glucopyranose units derived from bacterial digestion of starch (Szejtli, 1997). CDs can be cross-linked with suitable cross-linker agents (e.g., diacid chlorides, diisocyanates, and epichlorohydrin) to form 3D polymer host networks with variable functionality, solubility, and tunable inclusion properties (Karoyo and Wilson, 2013). Polymeric adsorbents that incorporate CDs as macromolecular porogens into the polymer network offer a promising approach for the sustainable removal of chlorophenols (Crini, 2003). **(**1) They are sustainable, scalable, and relatively low-cost, (2) display size-selective uptake toward target pollutants, and (3) adsorbent regeneration can be achieved without harmful solvents and reduced energy requirements due to the role of non-covalent adsorbent-adsorbate interactions. CD-based polymer adsorbents have favorable binding affinity with organic guests that afford surface immobilization of bacteria, thus offering dual function properties (adsorption and biodegradation) (Martins et al., 2013; Bosso and Cristinzio, 2014). The unique host-guest affinity of such adsorbents as CDs with various organic molecules (Palepu and Reinsborough, 1989; Melani et al., 1995; Wilson and Verrall, 1998; Tatsuno and Ando, 2006; Karoyo et al., 2011) is revealed by the range in stability constant values (*K*_i_; ca.10^2^-10^5^ M^-1^) for 1:1 complexes. *K*_i_ values are known to vary according to the hydrophile-lipophile balance (HLB) for guests such as phenols (Leyva et al., 2001) and perfluoroalkyl guests (Karoyo and Wilson, 2013, 2016b). Other support matrices for bacterial immobilization such as activated carbon (AC) and zeolites are used owing to their enhanced microbial thermostability and non-biodegradability (Rehm and Miinster, 1985; Annadurai et al., 2000; Quintelas et al., 2006). However, the reduced uptake selectivity imparted by such apolar surfaces limit their use in chemical separations (Bosso and Cristinzio, 2014). Biopolymer supports such as chitosan (Annadurai et al., 2000), polyurethane foams (Hu et al., 1994), and alginate (Abou Seoud and Maachi, 2003) have reduced mechanical stability and may undergo biodegradation in wastewater processing (Bosso and Cristinzio, 2014). Synthetic polymers such as polyacrylamide and PVC offer greater mechanical stability in wastewater treatment applications (Cassidy et al., 1996; Leenen et al., 1996) but are limited by their reduced pore structure and surface functionality. By contrast, reports on the use of CD-based polymers as immobilization supports for bacteria are sparse when compared with conventional composite materials. Pluemsab et al. have reported the use of α-CD cross-linked alginate beads as an immobilization support for the adsorption and *in situ* bacterial biodegradation of nonylphenol (Pluemsab et al., 2007). Similarly, Sevillano et al. used β-CD hydrogels as bacterial supports in a fluidized bed reactor system (Sevillano et al., 2008). More recently, Safont et al. (2012) have used β-CD polymer hydrogels to immobilize phenol-degrading microorganisms. Thus, cross-linked CDs offer a potential alternative as dual adsorbent/immobilization materials for biotechnology and environmental remediation (*cf*. Graphical Abstract) due to their tunable pore structure, surface area (SA), and surface chemical properties (Dhake et al., 2013).

**Graphical Abstract:**
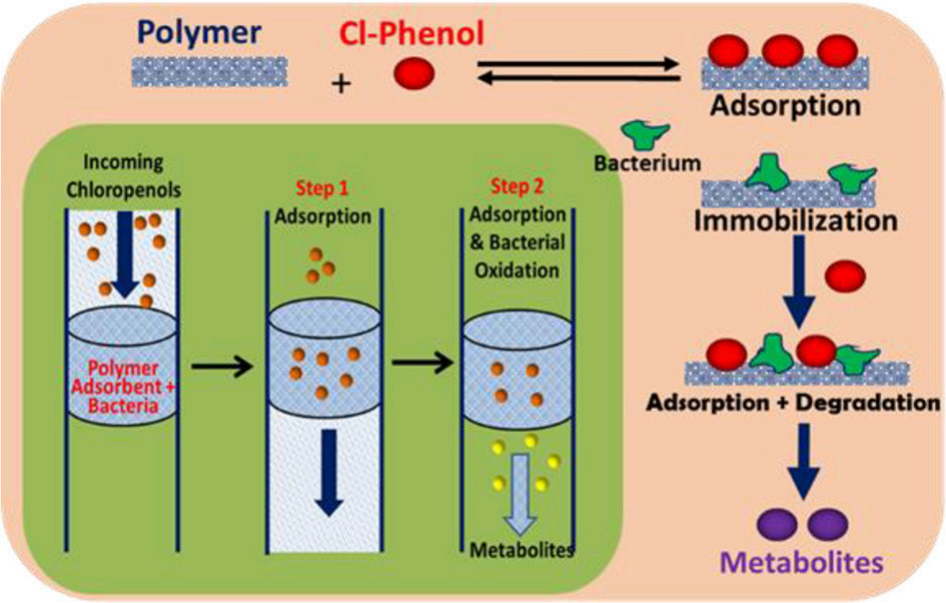
Schematic diagram of adsorption and *in-situ* biodegradation of pentachlorophenol on a polymer-immobilized bacteria system.

Herein, we report several cross-linked polymers that contain β-CD with variable mole ratios of cross-linker (hexamethylene diisocyanate; HDI), hereafter denoted as HDI-X; where X = 1, 3, and 6. The objectives of this work are manifold: (1) to evaluate the uptake and selectivity of the HDI-X polymers toward phenolic compounds; (2) to gain insight on the adsorbent-adsorbate modes of interaction; and (3) to evaluate the use of the HDI-3 polymer as a support for PCP-degrading microorganisms with dual function properties related to adsorption and *in-situ* biodegradation of PCP and its metabolites (*cf*. Graphical Abstract). Isotherm studies were used to screen the HDI-X polymers as potential adsorbents for PCP and its oxidative metabolites along with other phenolic guests [2,4,6-trichlorophenol (TCP); 2,4,6-trimethylphenol (TMP)] in aqueous solution. The adsorption results for the HDI-X biopolymers were compared against a commercial GAC adsorbent to study the role of textural and surface properties and to assess the selectivity of the polymer materials. The polymer and GAC adsorbents reveal variable selectivity toward the various phenolic compounds in this study as determined by electrospray ionization mass spectrometry (ESI-MS). 1D/2D ^1^H NMR spectral results of a soluble model HDI-1 polymer reveal that the mode of interaction between various HDI-1/adsorbate systems vary according to the sorption capacity and relative binding affinity. The adsorption and *in-situ* biodegradation properties of a selected polymer-immobilized *Sphingobium Chlorophenolicum* (SpC) system (HDI-3-SpC) with PCP and its reactive metabolites [6-dichloro-1,4-hydroquinone (DCHQ) and tetrachloro-1,4-benzoquinone (TCBQ)] were evaluated. The HDI-3-SPC (polymer-bacterium) system affords dual function (adsorption and *in-situ* biodegradation) of DCHQ and TCBQ metabolites. 2,6-dichlorophenol (2,6-DCP) was used as a model compound to support the uptake of DCHQ by the HDI-3 polymer. The apoptosis and cytotoxic effects of the PCP metabolites with various cancer cell lines reveal that exposure to PCP and/or its metabolites may induce or inhibit cancer cell deaths, further illustrating the need for effective and sustainable remediation methods for PCP and its metabolites. A key contribution of this study relates to the characterization of the adsorption properties of cross-linked polymer systems and their potential utility as bacterial supports for the *in-situ* removal and biodegradation of phenolic waterborne contaminants. The dual function properties of the polymer-bacterium system herein afford dual uptake and degradation of PCP and its oxidative metabolites such as DCHQ and TCBQ.

## Materials and methods

### Materials

Granular activated carbon (GAC; Norit Rox 0.8, VWR Canada Ltd.) was pre-treated by refluxing in methanol for 24 h at 70°C to remove any impurities. Pentachlorophenol (PCP; 97%), 2,4,6-trichlorophenol (TCP; 97%), 2,4,6-trimethylphenol (TMP; 99%), and 2,6-dichlorophenol (2,6-DCP) were purchased from Sigma Aldrich and were used as received. Hexamethylene diisocyanate (HDI), β-Cyclodextrin (β-CD), dimethyl acetamide (DMA), sodium hydroxide (NaOH), methanol, ammonium hydroxide (NH_4_OH), potassium hydrogen phosphate (KH_2_PO_4_), and 4Å (8–12 mesh) molecular sieves were purchased from Sigma-Aldrich Canada Ltd. (Oakville, ON). All materials were used as received unless specified otherwise.

### Apoptosis and cytotoxicity assays

Human breast cancer cell lines MCF7 (ER^+^, PR^+^, HER2^−^), Sk-Br-3 (ER^−^, PR^−^, HER2^+^), and MDA-MB-231 (ER-, PR-, HER2-) were purchased from American Type Culture Collection (ATCC). The cell lines were cultured in T-75 culture flasks under ATCC-recommended cell culture conditions at 37°C in a Forma™ Series II Water-Jacketed CO_2_ Incubator (ThermoFisher Scientific Inc., Waltham, MA, USA). Cell lines MCF7 and Sk-Br-3 were cultured with 5% CO_2_, while the cell line MDA-MB-231 was cultured with 0% CO_2_. Culture media were changed every 2–3 days for each cell line. The cultured breast cancer MCF7, Sk-Br-3 or MDA-MB-231 cells were plated in 96-well plates (10,000 cells/well) and grown to 70–80% confluence before being treated with DCHQ for 24 h. Treatment with DMSO, in which DCHQ stock solution was prepared, was used as negative control. Apoptosis (caspase 3/7 level) and cytotoxicity (lactate dehydrogenase level) were measured using the Promega Caspase-Glo® 3/7 Assay and the CytoTox96® Non-Radioactive Cytotoxicity Assay, respectively.

### Synthesis of the β-CD/HDI polymer adsorbents

The synthesis of the CD-based polymer adsorbents (HDI-1,−3, and−6) was adapted from a known method (Mohamed et al., 2011a). DMA was dried with 4Å (8–12 mesh) molecular sieves. The 1:1, 1:3, and 1:6 β-CD/HDI polymers (denoted HDI-1,−3, and−6) were prepared by adding 1 mmol equivalent of dried β-CD to a round bottom flask with stirring until dissolved in 10 mL of DMA, followed by the addition of 1, 3, or 6 equivalents of HDI in 30 mL of DMA to the reaction mixture. An illustration of the cross-linking reaction is reported elsewhere (see Scheme 1 in Mohamed et al., 2011a). The solution was stirred with heating at 68 ± 2°C for 24 h under argon and cooled to 23°C. The excess DMA was removed under vacuum (pressure ca. 1 mbar), followed by the subsequent addition of cold methanol (ca. 0°C) to the gelled product and filtration through Whatman no. 2 filter paper to obtain the crude solid product. The product was washed with methanol in a Soxhlet extractor for 24 h to remove unreacted reagents and low molecular weight oligomers. The product was dried in a pistol dryer for 24 h, ground into a powder, and passed through a sieve (size 40 mesh) to ensure a uniform particle size. A second cycle of washing in the Soxhlet extractor with anhydrous diethyl ether for 24 h was followed by drying, grinding, and sieving, as outlined above. The structure of HDI-X polymers was characterized using FT-IR/NMR spectroscopy, TGA, and CHN analyses, as reported elsewhere (Mohamed et al., 2011a). Table 1 lists some selected physicochemical properties of the cross-linked polymer materials (Mohamed et al., 2010, 2011a; Dhake et al., 2013; Karoyo and Wilson, 2016b).

**Table 1:** Physicochemical and surface properties of the phenolic Adsorbates and GAC/Polymer Adsorbents.

**Phenolic Adsorbates**	** 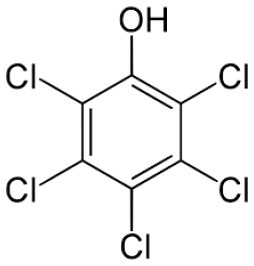 **	** 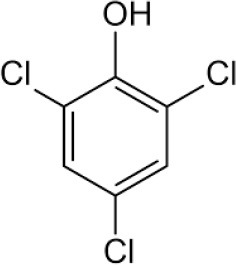 **	** 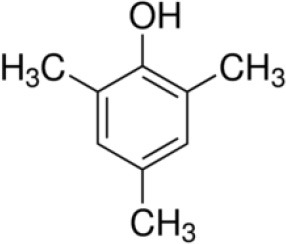 **	** 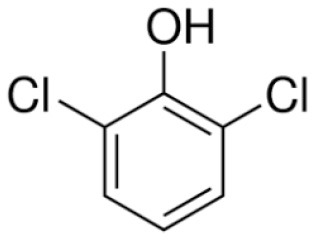 **
**Property**	**Pentachlorophenol, PCP**	**2,4,6-Trichlorophenol, TCP**	**2,4,6-Trimethylphenol, TMP**	**2,6-Dichlorophenol, DCP**
Physical state (EPA toxicity)	White crystalline solid (Class B2)	Crystalline granules (Class B2)	Brown crystalline solid	White crystalline solid
Molar mass (g/mol)	266.34	197.45	136.19	163.00
Boiling point (°C)	309	246	220	218–220
Melting point (°C)	190	64–66	70-72	64–66
Water Solubility (g/L @ 25°C)	0.02	0.8	1.01	0.5
Vapor pressure (mmHg @ 25°C)	0.0001	0.008	0.1	0.036
pK_a_ @ 25°C	4.5-4.7	6.23	10.88	6.8
K_ow_	5.05	3.69	2.73	2.64
**GAC AND POLYMER ADSORBENTS**				
	**Surface Area (m**^2^**/g)**	**Content of CD (mol %)**	Accessibility^*^(β-CD; %)	**Linker content (%)**
Norit GAC	1,100	–	–	–
HDI-1	<1	87.1	100	12.9
HDI-2	<1	69.2	4.78	38.7
HDI-3	<1	52.9	~0	77.4

* *Refers to values obtained from the reference by Mohamed et al. (2010)*.

### Adsorption isotherms

Adsorption isotherm experiments were conducted in 4 dram glass vials loaded with ca. 2 or 10 mg of adsorbent in 7 mL of solution containing variable phenol concentration [ca. 0.2–4 mM (or 8 mM)] in 0.1 M phosphate buffer at pH 9.0. The pH was adjusted using 1 M NaOH and the vials were sealed with paraffin film in a screw cap vial, where the contents were continuously agitated on a mechanical shaker at 150 rpm and ambient temperature (295 K). The equilibration times were maintained at 24 h. After the sorption process, the solutions were centrifuged and the residual equilibrium concentration of the supernatant was determined using a double beam spectrophotometer (Varian CARY 100) at 295 K by monitoring the spectral absorbance at the λ_max_. The molar absorptivity (ε) for each adsorbate at pH 9.0 was determined at variable wavelength conditions; ε_PCP_ = 5,100 M^−1^cm^−1^ (λ = 320 nm), ε_TCP_ = 4,830 M^−1^cm^−1^ (λ = 311 nm), and ε_TMP_ = 1454 M^−1^cm^−1^ (λ = 280 nm). Approximately 10–15 data points were obtained for each isotherm, where the residual adsorbate concentration was determined from the optical absorbance values at each λ_max_ for each phenol. Alkaline conditions were chosen throughout this study to ensure adequate solubility and to enable UV-vis spectral detection of the unbound adsorbate before and after adsorption. At pH 9.0, the adsorbates exist as anion species since the pH conditions lie above the pK_a_, according to values in Table 1 for each phenol except TMP (pK_a_ = 10.9).

### Mass spectrometry (LC-ESI-MS)

The percent uptake ((C_0_-C_e_)/C_0_)^*^100) and selectivity of the various adsorbents for the phenolic compounds in mixtures of equimolar solutions was evaluated using ESI-MS. Ten milligram per liter mixed phenol solution was prepared by dissolving equivalent weight amounts of each sorbate (PCP, TCP, and TMP) in Millipore water, where the pH was adjusted to 9.0 using NH_4_OH solution. Seven milliliters of the mixed solution was added to vials containing ca. 20 mg of β-CD, HDI-3, HDI-6, or GAC. The vials were sealed with paraffin film and were continuously agitated on a mechanical shaker (~150 rpm) for 24 h to achieve equilibrium. Thereafter, the solutions were centrifuged and the values of C_e_ were determined using a hybrid quadrupole-TOF liquid chromatography (LC) ESI-MS and compared with the initial concentration (C_o_) of the components. The ESI spectra were acquired in the negative ion mode and LC separations were performed with a QStar XL System (AB Sciex Instruments) using methanol and water containing 0.1% NH_4_OH as the eluent.

### Bacterium immobilization

*Sphingobium chlorophenolicum* (SpC) was obtained from the American Type Culture Collection (ATCC 53874; Manassas, VA, USA) and was cultured in Laura-Bertani (LB) media (10 mL) in a sterile 15 mL culture tube at 37°C. The bacterial growth rate was evaluated by monitoring the absorption at 600 nm (OD600). As the OD600 reached 0.6, the bacterial cells were used as required. Bacterium (SpC) immobilization onto HDI-3 polymer was achieved by equilibrating, through gentle agitation, a known concentration (~ 100 μL) of the cells in 1X phosphate-buffered saline (PBS) solution (pH 8.0) with ca. 20 mg of the polymer for 2 h at 295 K. The viability of a CD-based polymer (HDI-3) as a bacterial-support was underscored in the previous sections. Additionally, cell immobilization techniques onto a solid support via simple adhesion/adsorption methods are well known (Woodward, 1988; Elakkiya et al., 2016), To independently evaluate whether the HDI-3 polymer had suitable immobilization/adhesion affinity for the bacterial cells, the polymer was coated onto microscope glass slides via drop-casting. Images of the immobilized polymers were taken at 25 × magnification using a Renishaw Raman InVia Reflex SOP Microscope with a visible light source (REO4; Smiths Illuminator II). To the polymer-coated microscope slides, 100 μL of SpC was added onto the polymer surface. Excess culture media was removed by wicking excess solvent using a Whatman no. 2 filter paper. Thereafter, the glass slides were air-dried at 295 K prior to recording images of immobilized cells. Finally, the immobilized bacteria were washed with Milli-Q water thrice to examine the adsorption affinity of the bacterial cells onto the polymer film. Excess water was removed using filter paper, as described above, followed by air-drying.

### *In-situ* adsorption and metabolism of PCP/DCP

A one-pot experiment (Mohamed et al., 2015) was used to evaluate the activity and contribution of HDI-3 to PCP biodegradation by SpC. The bacterium cells (with or without HDI-3) were added to sealed dialysis bags and equilibrated in a 1X PBS buffer (pH 8.0) for 24 h. The dialysis membrane and contents were then submerged in beakers containing ca. 100 ml of PCP solution (0.2 mg/L). The system was stirred at 20 rpm, where 100 μL aliquots of the PCP solution were drawn from each system, containing polymer (and without polymer) at 1 h intervals. The degradation rate of PCP was evaluated by monitoring the absorbance (λ_max_ 320 nm) of the sample aliquots at variable time intervals. The uptake properties of PCP and 2,6-dichlorophenol (DCP; λ_max_ 285 nm; model compound for DHCQ) with HDI-3 were evaluated vs. time in the one-pot setup (*cf*. **Figure 3**), independently. The residual amounts of PCP and/or its metabolites, and DCP were measured using a double beam spectrophotometer (Varian CARY 100) at 295 K by monitoring the changes in optical absorbance at the λ_max_.

### Models and equations

The heterogeneous adsorption isotherms are shown as equilibrium uptake of the adsorbate by the adsorbent polymer phase (Q_e_; mmol/g or mg/g) vs. the residual equilibrium solution concentration of the adsorbate (phenol) species (C_e_; mM or mg/l) by Equation (1). C_o_ is the initial adsorbent concentration (mM), *m* is the mass of adsorbate (g), and V is the volume of the solution (L).

(1) $$\begin{array}{r} Q_{e}=\frac{\left(C_{o}-C_{e}\right)\times V}{m} \end{array}$$

The isotherm results were fitted using the Sips model (Sips, 1948), described by Equation (2), where K_s_ (L/g) is the equilibrium sorption constant, Q_m_ (mmol/g) is the monolayer sorption capacity of the adsorbate, and n_s_ is the isotherm model exponent term that represents the degree of heterogeneity of the sorbent surface.

(2) $$\begin{array}{r} Q_{e}=\frac{Q_{m}{\left(K_{s}C_{e}\right)}^{n_{s}}}{1+{\left(K_{s}C_{e}\right)}^{n_{s}}} \end{array}$$

### NMR spectroscopy

All ^1^H NMR experiments were performed using a 2-channel Bruker Avance (DRX) spectrometer operating at a ^1^H resonance frequency of 500.13 MHz. The polymer/phenol samples were prepared in D_2_O at ca. pH 9 (adjusted using NaOH). HDI-1 was used as a soluble model polymer for the solution NMR study. The low water solubility of HDI-3 and−6 polymers precluded their analysis in solution. All ^1^H NMR spectra were referenced externally to tetramethylsilane (TMS, δ = 0.0 ppm) with a recycle delay of 2 s and a 90° pulse length of 10 μs. For all 2-D rotating-frame Overhauser effect spectroscopy (ROESY) experiments, the spin-lock times were maintained at 350 ms. The spectra were acquired with a spectral width of 10 ppm in 1 k data points (2-D gROESY) and 8 scans. The spin-lock power levels for the 2-D ROESY were set to 21.33 dB and the NMR spectra were acquired at 295 K.

## Results and discussion

### Adsorption and uptake selectivity of HDI-X polymers

**Figure 1:**
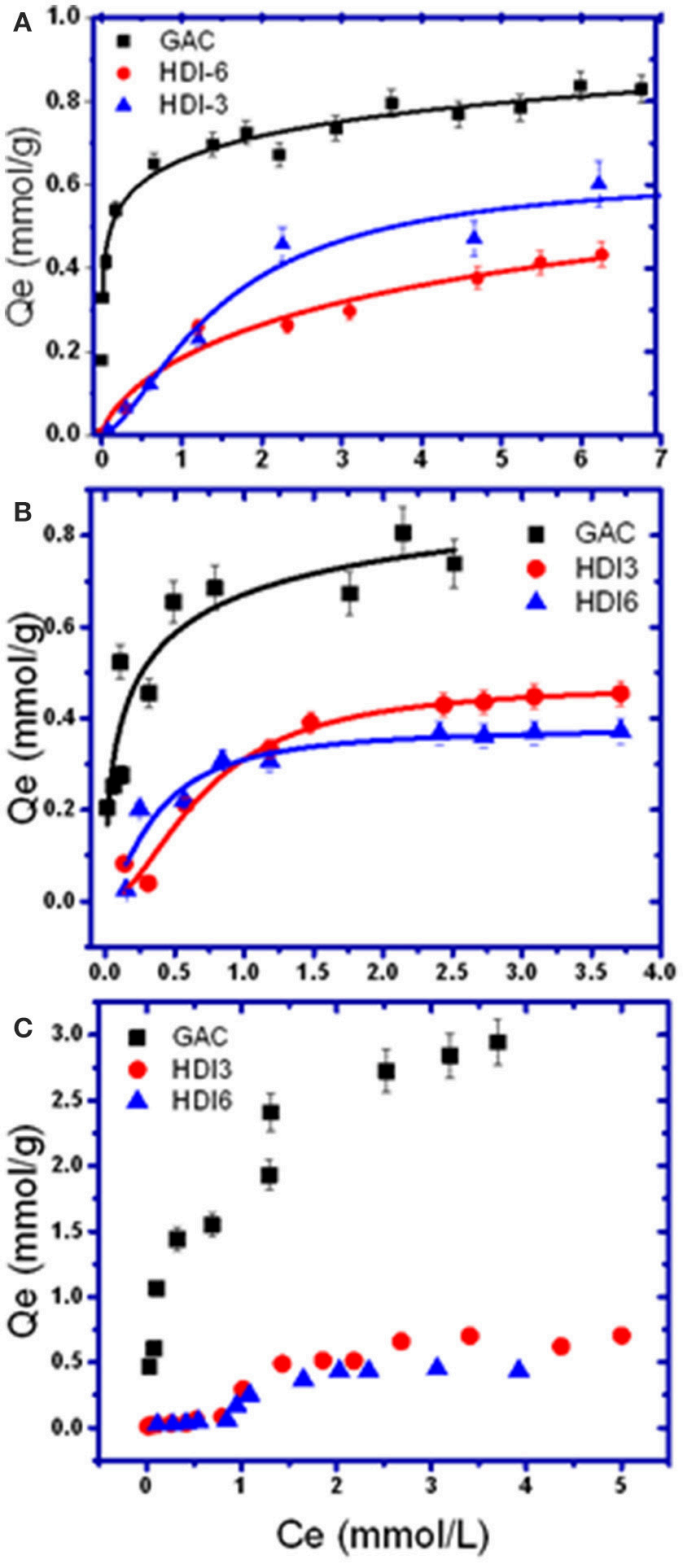
Sorption isotherm models for **(A)** pentachlorophenol (PCP), **(B)** 2,4,6-trichlorophenol (TCP), and **(C)** 2,4,6-trimethylphenol (TMP) with various adsorbents.

The adsorptive uptake properties for PCP with the insoluble HDI-X polymers (HDI-3 and−6) at ambient pH and 295 K were compared with a commercial GAC adsorbent (Figure 1A). Results for other phenols were also compared as shown in Figures 1B,C. Note that the adsorption of HDI-1 was not examined because this polymer is soluble in water at the specified conditions and does not satisfy the requirements for heterogeneous adsorption in Equation (1). The experimental results were well-described by the Sips model (Equation 2) with R^2^ ≈0.92–0.98, where the best-fit parameters are listed in Table 2. In Figure 1, Q_e_ increases monotonically as C_e_ increases and levels off at C_e_ ≈ 1.0 mM for the various adsorbent-adsorbate systems. The sorption isotherm of GAC/PCP system (Figure 1A) displays an H-type curve with high affinity (Giles et al., 1960), with a Q_m_ value ca. 1.0 mmol/g that reaches saturation for C_e_ ≈ 1.0 mM. The high PCP uptake with GAC herein is supported by the higher K_s_ value (*cf*. Table 2). In contrast, the GAC/TCP system (Figure 1B) displays a Langmuir-type isotherm (Giles et al., 1960) with a higher Q_m_ value (1.43 mmol/g) that corresponds with a reduced value of C_e,sat_ ≈0.5 mM. TMP shows high affinity for GAC with a notable value of Q_m_ (2.38 mmol/g) relative to TCP (1.43) and PCP (1.01). The sorptive uptake of the phenols by the HDI-X polymers are comparable, where the Q_m_ values of HDI-3 are slightly greater than those of HDI-6 with generally lower K_s_ values.

**Table 2:** Sips isotherm model best-fit parameters for various adsorbent-adsorbate systems.

	**Q_m_ (mmol/g)**	**K_s_ (L/g)**	**K_eq_ (L/mol)**	**n_s_**	***R*^2^**
**PCP**					
GAC	1.01 ± 0.08	7.64 ± 1.01	2.01	0.35	0.98
HDI-3	0.50 ± 0.04	0.54 ± 0.05	0.14	1.27	0.96
HDI-6	0.58 ± 0.06	0.81 ± 0.07	0.22	1.93	0.98
**TCP**					
GAC	1.43 ± 0.10	0.84 ± 0.09	0.17	0.48	0.93
HDI-3	0.52 ± 0.04	1.73 ± 0.20	0.34	1.42	0.98
HDI-6	0.45 ± 0.03	2.39 ± 0.31	0.47	1.93	0.98
**TMP**					
GAC	2.38 ± 0.03	5.95 ± 0.09	0.81	0.54	0.92
HDI-3	0.67 ± 0.03	0.57 ± 0.05	0.08	2.82	0.98
HDI-6	0.48 ± 0.02	1.05 ± 0.04	0.14	5.78	0.92

The uptake profiles of the various adsorbent materials with the phenolic adsorbates in Figure 1 highlight the role of the textural and surface properties in the sorption process. Other molecular properties of the adsorbates, such as polarizability and size, determine their affinity with the adsorbents via surface interactions, along with intra-particular diffusion within the pores of the adsorbents. The physicochemical properties of the various adsorbents and adsorbates are listed in Table 1. The sorption capacity of the GAC/PCP system (Q_m_; 1.01 mmol/g) herein agrees closely with another report according to a range of Q_m_ values between 1.5 mmol/g (Hameed and Rahman, 2008) and 0.7–1.1 mmol/g (Mollah and Robinson, 1996), where the uptake values vary as a function of pH and the surface properties of GAC. Wilson et al. (2011) reported a lower value at Q_m_ (0.306 mmol/g) for GAC in line with its lower SA. In contrast, Q_m_ values reported for the GAC/TCP system typically range between 0.13 and 2.5 mmol/g (Hameed et al., 2008; Fan et al., 2011). Despite the greater sorption of TCP (1.43 mmol/g) relative to PCP, a much greater K_s_ value was noted for the GAC/PCP system. The latter relates to the complementary apolar nature of GAC and PCP (*cf*. Table 1) that favor apolar adsorbent-adsorbate interactions. In addition to the apolar surface of GAC, it is noteworthy that the greater Q_m_ values also correspond to its greater SA (*cf*. Table 1). In the case of the HDI-X polymers, the observed trends in the uptake of PCP (HDI-6 > HDI-3) and TCP (HDI-3 > HDI-6) relate to the relative molecular size of the phenol adsorbates. Different modes of interaction are likely to occur for PCP and TCP with the adsorbents according to size and electronic effects that stem from variable chloro-substituent effects. The reversal of trends can be understood in terms of the *size-fit* considerations for such host-guest systems and the occurrence of binding interactions at the inclusion and/or non-inclusion sites, as supported by the disparity of the K_s_ values in Table 2 and the NMR results (*vide infra*). The Q_m_ values of the HDI-X/phenol systems lie within range of reported values (0.054–1.17 mmol/g) for uptake of PCP and phenols such as 2,4-dichlorophenol(2,4-DCP) with CD-based urethane polymers (Wilson et al., 2011). The Q_m_ values for the polymer/TCP systems are variable: 1.2 mmol/g (pH 4) for molecularly imprinted polymers (MIPs) (Pan et al., 2011) to 0.8 mmol/g (pH 7) for β-CD polymers (Li et al., 2012), in agreement with results obtained herein. The uptake properties of TMP by the adsorbents generally exceed those for PCP and TCP. More recently, Dabrowski et al. (2005) reviewed the uptake properties of GAC with phenols. The Q_m_ values for carbonaceous adsorbents such as blast furnace dust and sludge, along with synthetic polymers that range from 0.3 to 0.7 mmol/g (Jain et al., 2002; Ardelean et al., 2012). The greater Q_m_ values noted for TMP with GAC or HDI-X polymers herein relate to the unique polarizability and size effects of the methyl-groups. This is further supported by unique sorption isotherms for the HDI-X/TMP system (*cf*. Figure 1C) where the isotherms converge with the Sips model over a limited concentration range (up to C_e_ ≈ 4 mM). The best-fit parameters listed in Table 2 and the trends in Figure 1C indicate that multilayer adsorption occurs for the polymer/TMP systems, according to the rise in uptake at higher C_e_ values in the post-plateau region. This is in accordance with multilayer profiles reported by Giles and Hassan for the adsorption of organics by chitin biopolymers (Giles and Hassan, 2008). The modes of interaction for the complexes of the various adsorbent/phenol systems were further studied by using 1D/2D NMR spectroscopy.

**Figure 2:**
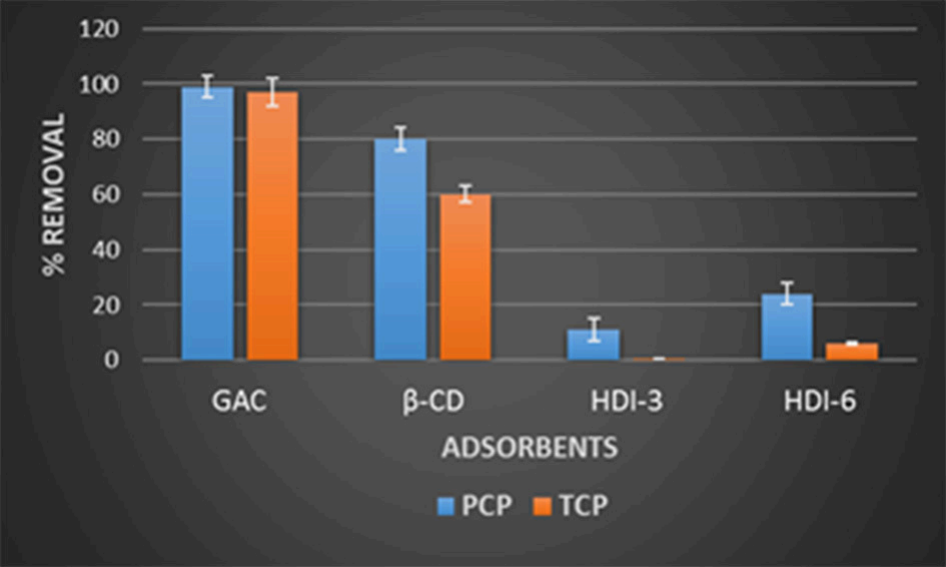
Selective uptake of the various adsorbents in solutions containing equimolar amounts of pentachlorophenol (PCP) and 2,4,6-trichlorophenol (TCP).

**Figure 3:**
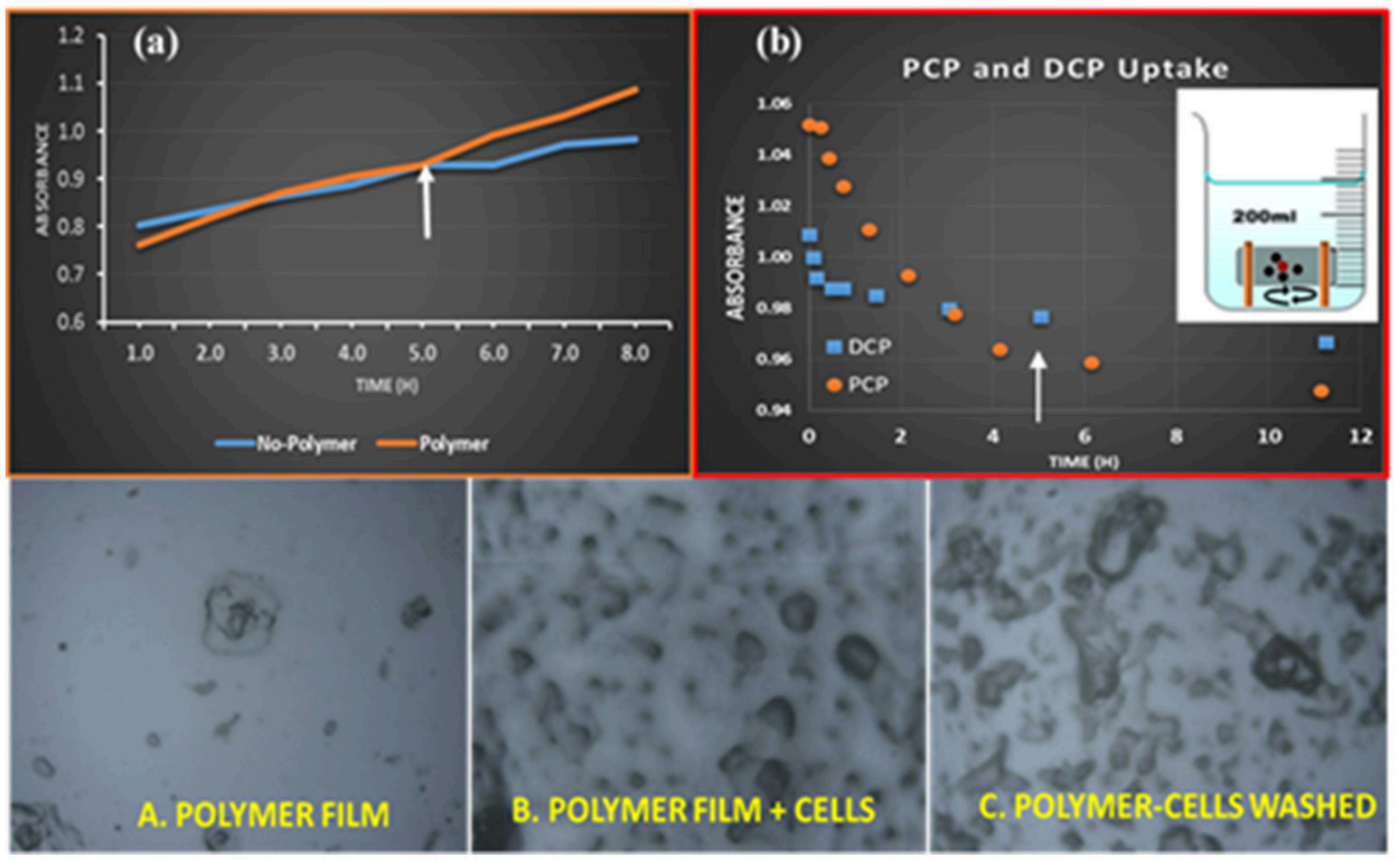
**(a)** *in-situ* bacterial decomposition of PCP in the presence and absence of HDI-3, **(b)** Kinetic uptake of PCP and DCP by HDI-3, **(c)** Optical microscopy images of HDI-3 with **(A)** no cells, **(B)** immobilized cells, and **(C)** immobilized cells after successive washes with a pH 7 buffer.

The molecular selective uptake of the polymer adsorbents (HDI-3 and HDI-6) with equimolar phenol mixtures (PCP and TCP) was studied using LC-ESI-MS, and compared against that for GAC and native β-CD (*cf*. Figure 2). Note that the ESI-MS of TMP was not observable under the given experimental conditions and may be due to its low ionization yield, in accordance with its lower K_a_ value (*cf*. Table 1). Thus, the results for TMP are not shown. The negative ions at *m/z* 265 and 197 amu were used to estimate the concentration profiles of PCP and TCP, respectively. In Figure 2, nearly quantitative removal of the phenols (PCP and TCP) by GAC (ca. 100%) occurs for these conditions, in accordance with its high SA (*cf*. Table 1). Correspondingly, lower values were noted for β-CD (~80%) and the HDI-X polymers in line with SA effects. The selective removal (%) of TCP and PCP with the various adsorbents are listed: GAC (≈97 and 99) > β-CD (≈60 and 80) > HDI-6 (≈6 and 24) > HDI-3 (≈0.6 and 11). The lower uptake (%) for the HDI-X polymers relative to β-CD relates to steric effects and configurational entropy of the polymer that affect efficient binding of the phenol within the apolar cavity of β-CD (Mohamed et al., 2010). The equilibrium sorption studies above provide insight on the uptake properties of adsorbents. On the other hand, molecular selective uptake/removal (S_R_) property is a key feature of support materials if they are to be considered as viable matrices for microorganisms. This is especially true when the uptake of specific target pollutants is desired. In Figure 3, GAC displays a uniform uptake for PCP (ca. 99%) and TCP (ca. 97%), indicating a low molecular selectivity between PCP and TCP by a low S_R_ value of ca. 2%. The low molecular selectivity for GAC relates to the role of non-specific hydrophobic effects. By contrast, the CD polymers (HDI-3 and−6) possess greater molecular selectivity; S_R_ ≈75% (HDI-6) and S_R_ ≈ 95% (HDI-3). The contribution of host-guest interactions at the inclusion and/or non-inclusion binding sites are anticipated for the polymers that vary according to the level of cross-linking. This is revealed by the improved selectivity for pure β-CD (S_R_ ≈ 25%). In addition to its insolubility in water, HDI-3 has properties of a suitable support material for bacterial immobilization due to its superior selectivity and high sorption capacity relative to HDI-6 and GAC.

### Immobilization of bacterium onto HDI-X polymers

Based on the adsorption properties and the molecular selective uptake results for the various polymer/phenol systems noted above. Thus, HDI-3 was chosen for further study as the immobilization support for *Sphingobium Chorophenolicum* (SpC). The immobilization of SpC bacterium cells onto the HDI-3 solid support was described in the experimental section. Also, the degree of adsorption of the bacterium cells was tested on HDI-3 films that were cast onto glass slides (Karoyo and Wilson, 2016a) by use of optical microscopy (*cf*. Figures 3A–C and Experimental Section). According to the microscopy results in Figure 3, SpC remained immobilized onto the polymer surface films after 3 successive cycles of washing with a buffer. Therefore, the SpC cells were adsorbed favorably onto the surface sites of HDI-3 since washing with aqueous buffer did not elute the cells to any notable extent, further indicating the stability of the immobilized biofilm system (*cf*. Figures 3A–C). The favorable immobilization and adsorption properties of HDI-3 relate to its moderate SA, pore structure properties, and presence of hydrophilic groups (*cf*. **Figure 5**) such as -OH and -NH. The immobilization stability of the HDI-3/SpC system shows parallel results for the efficacy of HDI-X materials as immobilization supports for a lipase enzyme/polymer system reported elsewhere (Dhake et al., 2013) along with other types of CD-polymer/bacteria systems reported (Pluemsab et al., 2007; Safont et al., 2012).

### Adsorption and metabolism of PCP using HDI-3/SpC composite materials

The relative contributions to adsorption and metabolism of PCP using the HDI-3-SpC system require a comparison of the activity of SpC in the absence and presence of the HDI-3 polymer. To this end, a one-pot adsorption method (*cf*. Figure 3b, Inset) was employed where the activity of SpC toward PCP was compared at two conditions: “No-Polymer,” where SpC is in its unbound state with HDI-3 polymer and “Polymer,” where SpC is in its bound state with HDI-3 (*cf*. Figure 3a). The initial activity of SpC in its immobilized state (“Polymer”) and dispersed form without polymer (“No-Polymer”) is considered insignificant up to 5 h that may relate to a kinetic induction period. Greater activity was observed at longer time (t > 5 h) for the immobilized polymer form as denoted by the arrow in Figure 3a. In Figure 3a, the observed increase in absorbance relates to the formation of various PCP metabolites such as DCHQ and TCBQ, as described elsewhere (Ling et al., 2016). The degradation pathway of chlorophenols and other quinones to intermediates/byproducts such as DCHQ and TCBQ with the use of SpC and other microorganisms have been reported (Arora and Bae, 2014; Lopez-Echartea et al., 2016). Herein, the PCP metabolic by-products were monitored according to the measurement of absorbance values (λ_max_ = 340 nm). In Figure 3a, the slopes for each profile at t > 5 h are listed in parentheses for the immobilized SpC (“Polymer”; 0.0516) and non-immobilized SpC (“No-polymer”; 0.0207), where the offset relates to an overall difference that exceeds 100% for the immobilized vs. non-immobilized form of SpC bacteria. To further establish the source of the difference in activity between the “No-polymer” and “Polymer” conditions, and to support the role of synergism for the bacteria in the adsorption and/or biodegradation of PCP by HDI-3-SpC, an independent adsorption isotherm for PCP and its model metabolite by-product (DCP) was obtained, as shown in Figure 3b. According to the isotherm profile, the HDI-3/PCP system achieves a dynamic equilibrium between 4 and 5 h (see arrow in Figure 3b). Thus, the enhanced metabolism for HDI-3/SpC at t > 5 h in Figure 3a relates to the activity of the immobilized bacteria. The role of adsorptive uptake of HDI-3 can be ruled out since adsorption reaches a dynamic equilibrium prior to the end of the induction period. In the case of DCP (Figure 3b), the polymer reaches dynamic equilibrium sooner (t < 2 h) and indicates that polymer adsorption of the PCP metabolites may occur sooner if they are present in the medium. The enhanced biotransformation of PCP is inferred to arise from synergistic effects due to SpC-polymer immobilization.

### NMR studies of the sorptive interactions

The utility of NMR for the characterization of host-guest complexes in solution and the solid state is well-established (Pessine et al., 2012). 1-D NMR complexation-induced chemical shifts (CIS) and 2-D NOE (Nuclear Overhauser effects) allow for the characterization of the host-guest geometry via *through-space* dipolar interactions that lie within 3–5 Å (Bax and Davis, 1985). In the case of adsorbent-adsorbate systems, dipolar interactions provide strong support that non-covalent complexes are formed in a solvent medium (Wilson and Guo, 2012; Karoyo and Wilson, 2016b). Herein, HDI-1 was chosen over the other polymers (HDI-3 and−6) due to its water solubility and as a single phase model system to study the modes of interaction with TCP and TMP adsorbates in solution. Note that PCP was precluded from this study because the adsorbate is less amenable to ^1^H NMR spectral measurements in its ionized form.

**Figure 4:**
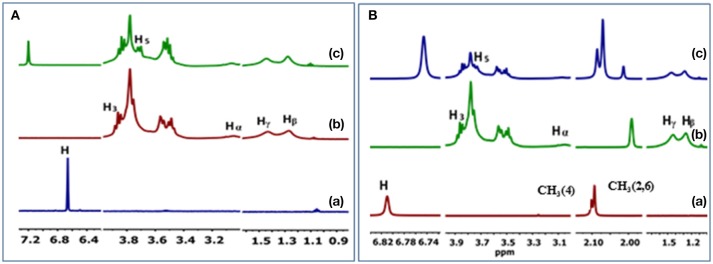
^1^H NMR spectral results for the complexes of HDI-1 polymer with **(A)** TCP and **(B)** TMP. (a) unbound guest, (b) unbound HDI-1, and (c) HDI-1/guest complex.

**Figure 5:**
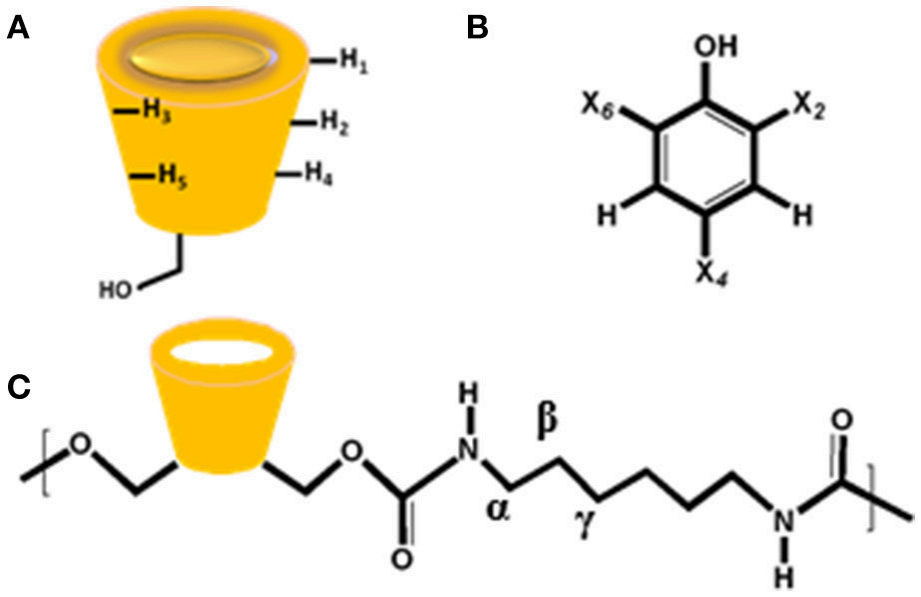
Structures of **(A)** β-Cyclodextrin (β-CD), **(B)** phenolic adsorbates (X = Cl or CH_3_), and **(C)** HDI-X polymer fragment, where X = 1 denoted above.

The ^1^H NMR spectra and CIS values for the HDI-1/TCP and HDI-1/TMP systems are shown in Figure 4 and Table S1, respectively. The resonances were assigned in accordance to a previous report (Karoyo and Wilson, 2016b) using the structural designation shown in Figure 5. The CIS values were computed as Δδ (ppm) = δ_bound_ – δ_free_. The two adsorbent-adsorbate systems display variable CIS effects for the phenolic protons; high field (+0.54 ppm) for TCP, and low field (−0.07 ppm) for TMP (*cf*. Table S1). The trends suggest greater deshielding for TCP that may indicate variable binding geometry for the inclusion vs. non-inclusion sites of HDI-3. The trend in CIS values for the linker domains (H_α_, H_β_, and H_γ_) reveal greater non-inclusion binding for TMP as indicated by greater deshielding of these nuclei due to inductive effects upon formation of non-covalent complexes. The reduced deshielding of similar linker nuclei for the HDI-1/TCP system is consistent with greater inclusion binding of the adsorbate. The inclusion binding of TCP with HDI-1 is supported by the greater value of K_s_ (*cf*. Table 2, Isotherm parameters). Inclusion binding is anticipated to be more favored over the non-specific binding at the interstitial domains. The separation of the intracavity H_5_ resonance for the HDI-1/TCP system further supports an inclusion mode of binding (*cf*. Figure 5A), where the guest (*cf*. Figure 5B) interacts with the narrow annular rim via the phenolic -OH group. A host-guest geometry of this type is consistent with the deshielding effect of the phenolic -OH group. In the case of the HDI-1/TMP system, binding at the inclusion cavity sites and non-inclusion domains can be inferred from the CIS values of the phenolic -OH, -CH_3_ of TMP and the -(CH_2_)_6_- groups of HDI, respectively (*cf*. Figure 5C). The 1D ^1^H NMR results above concur with the 2D ROESY results (Figure S1) that provide unequivocal evidence for the sorption of the adsorbates within the inclusion and at non-inclusion sites. In particular, TMP shows prominent interactions with both the cavity sites and the interstitial/non-inclusion active sites, in agreement with the 1D NMR results herein.

### Adsorbent-adsorbate interaction modes

The sorption properties of the CD-based polymers and GAC depend on several factors: (i) the nature of the adsorbent material (textural and surface chemical properties), (ii) adsorbate properties (e.g., polarizability, log K_ow_, and molecular size), and (iii) isotherm conditions (e.g., temperature, pH, ionic strength, and adsorbate concentration). According to the NMR results herein and a previous report (Karoyo and Wilson, 2016b), HDI-X polymers may adsorb phenolic species at dual (inclusion and non-inclusion) sites. The inclusion cavity sites in CD-based materials are primary adsorption sites at low to moderate cross-linking (Mohamed et al., 2011b). By contrast, the non-inclusion domains are secondary adsorption sites that may display greater affinity when the inclusion sites are sterically restricted due to excessive cross-linking (Mohamed et al., 2011b). The sorption of the smaller TCP molecule can be inferred to occur via inclusion binding as the predominant uptake mechanism, as evidenced by the higher K_s_ values (*cf*. Table 2) and supported by 1D/2D ^1^H NMR spectral results. The greater affinity between HDI-X and TCP relate to the apolar character of the adsorbate and the CD cavity inclusion sites, in line with the CIS values (*cf*. Table S1). In the case of the HDI-X/PCP system, interactions of PCP with non-inclusion sites contribute significantly to the overall binding process. Interactions at the linker domains are anticipated to be weaker (secondary) compared to the CD cavity sites, as evidenced by the offset in the K_s_ values for the TCP and PCP guest systems in Table 2. PCP has restricted binding at the CD inclusion sites because of its greater steric hindrance relative to TCP (Paleologou et al., 1990).

Based on the 1D/2D NMR results, stronger dipolar interactions occur for TMP at the inclusion and non-inclusion sites, as compared to the chlorophenols. This observation is consistent with the greater Q_m_ and K_s_ values of the HDI-X/TMP system, in agreement with the unique apolar nature of TMP. The NMR and isotherm results herein provide a basis to compare the chlorophenols (TCP and PCP) and TMP in terms of the *size-fit* complementarity with the CD cavity, along with the substituent effects of the guest. The -Cl and -CH_3_ groups have similar van der Waals volumes but differ according to inductive effects that vary based on the substitution pattern of the phenol and the substituent effects for the -Cl and -CH_3_ groups. TMP differs from PCP and TCP guests as shown by its greater acidity relative to the other chlorophenols: PCP (pK_a_ 4.7), TCP (pK_a_ 6.2), and TMP (pK_a_ 10.9). The greater pK_a_ of TMP contributes to its apolar character and favorable complex formation with the CD inclusion sites due to hydrophobic effects, even at pH 9. TMP has negligible ionization relative to TCP and PCP, where the reduced ionization of TMP governs its variable kinetic and equilibrium uptake properties (Sathishkumar et al., 2009). The higher SA of GAC materials and the role of non-specific binding relate to its greater sorptive uptake. The SA for GAC (SA ≈ 10^3^ m^2^/g) is two orders of magnitude greater than the CD polymers (SA ≈ 1 × 10^1^ m^2^/g) as listed in Table 1. Therefore, the greater Q_m_ values observed for the GAC/phenol systems largely relate to SA effects and the apolar character of the phenols that favor adsorption via hydrophobic effects (Blokzijl and Engberts, 1993). The role of pH and temperature conditions affect the sorption of organic guests as they modify the zeta-potential (Dabrowski et al., 2005; Liu et al., 2010; Li et al., 2012) and ionization properties (Sathishkumar et al., 2009) of the adsorbent-adsorbate system. The surface of GAC (Norit ROX 0.8; pH_pzc_ 7.3) (Kwon et al., 2014) has a slight negative charge at pH 9.0 and has Lewis base character. The chlorophenols exist as phenolates when the solution pH is above the pK_a_ and electrostatic repulsions lower the sorption capacity of the GAC adsorbent. In contrast, the greater pK_a_ of TMP affords favorable electrostatic interactions between the non-ionized adsorbate and the GAC adsorbent. In the absence of ionization (when pH < pKa), the phenyl ring of PCP has notable apolar character among the phenols, in agreement with its greater molecular weight and K_ow_ (*cf*. Table 1).

Based on the above discussion, the sorption of phenols with HDI-X polymers occur at the inclusion and/or non-inclusion sites based on the adsorption site accessibility and *size-fit* complementarity between the guest and the host sorption sites. Non-covalent interactions (H-bonding, van der Waals, and hydrophobic effects) are involved in the formation of the adsorbate-adsorbent complexes. In the case of GAC materials, the adsorption of phenols is strongly influenced by the build-up of surface charge at the sorbent interface in accordance with the pH and ionic strength of the solvent medium. The sorption of phenols onto the GAC and HDI-X polymer surfaces can be accounted for by the formation of electron donor-acceptor (EDA) complexes, π-π/CH-π dipolar interactions, and solvent effects (Mattson et al., 1969; Moreno-Castilla, 2004; Liu et al., 2010). EDA complexes occur between an aromatic ring of the phenol/polar functional groups (electron donor) of the adsorbate and with Lewis acid sites of an adsorbent (electron-acceptor). By contrast, the π-π mechanism involves interactions between the π electrons of phenyl rings and Lewis acid domains of the adsorbent that favor charge transfer, dispersive forces, and electrostatic interactions (Mattson et al., 1969). The magnitude of these π-π or C-H- π interactions are commensurate with the number and nature of substituent groups where -Cl is electron withdrawing, and -CH_3_ is an electron donor group (Cozzi et al., 1993).

### Toxicological assays

As mentioned previously, PCP is genotoxic and a potential carcinogen, where its toxicity is related to the formation of highly reactive metabolites (e.g., TCBQ and DCHQ) (Ling et al., 2016). The cytotoxicity assays of hydroquinones are well known in the literature (Li et al., 2015). Herein, the apoptosis (caspase 3/7 level) and cytotoxicity (lactate dehydrogenase level) of DCHQ toward human breast cancer cells were measured with the Caspase-Glo® 3/7 Assay and the CytoTox96® Non-Radioactive Cytotoxicity assay from Promega Corporation (Madison, WI, USA), respectively, using published protocols (Ling et al., 2016). DCHQ rapidly forms a stable semi-quinone radical in aqueous solution that induces cell apoptosis and DNA fragmentation in the body. Thus, the effect of the exposure of PCP to progression of cancer warrants a study of the apoptotic and cytotoxic effects of the metabolites with various cancer cell lines. Such a study would yield valuable toxicological information that could be used to develop improved breast cancer treatments and development of adsorptive materials as removable implants to separate the reactive metabolites from the tissues. The HDI-X-SpC dual-function system described herein offers tandem adsorption and *in situ* metabolism of PCP and its oxidative by-products. Thus, this system has promising potential for reducing exposure to PCP and other polychlorophenols.

**Figure 6:**
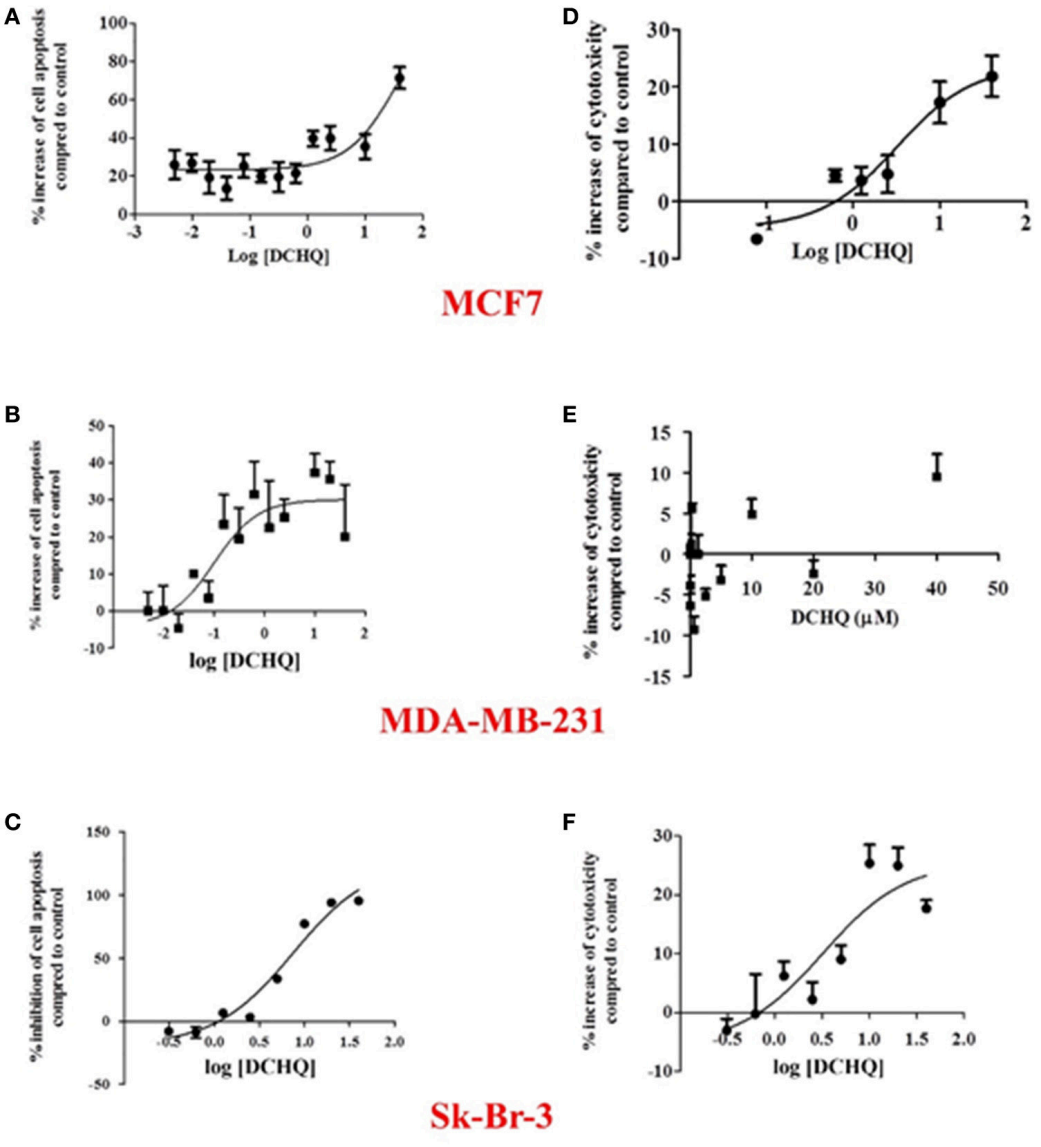
The apoptotic and cytotoxic effects of DCBQ toward: **(A,D)** human luminal A type MCF7, **(B,E)** triple-negative type MDA-MB-231, and **(C,F)** HER2 type Sk-Br-3 breast cancer cells, respectively.

The apoptotic and cytotoxic effects of DCHQ toward three different types of breast cancer cells (luminal A type MCF7 cells, HER2 type Sk-Br-3 cells, and triple-negative type MDA-MB-231 cells) are shown in Figure 6. According to Figure 6, DCHQ induced cell apoptosis in cell lines MCF7 (a) and MDA-MB-231 (b) as evidenced by an increase (%) of cell apoptosis as a function of DCHQ concentration. On the contrary, DCHQ inhibited cell apoptosis in the cell line Sk-Br-3 (c), in agreement with the monotonic increase of the inhibition (%) of cell apoptosis as a function of DCHQ concentration. Moreover, DCHQ exhibited cytotoxic effects toward MCF7 (d) and Sk-Br-3 (f) cells after a 24 h exposure. The significantly low cytotoxicity of DCHQ toward MDA-MB-231(e) implicated that the cytotoxic effect of DCHQ toward MDA-MB-231 likely occurs via apoptosis rather than necrosis, where longer treatment is needed to establish the apoptotic activity. In summary, DCHQ can enhance cell apoptosis in human luminal A and triple-negative types of breast cancer cells but result in a decrease in cell apoptosis for human HER2 type of breast cancer cells. Removable tumor-surface implants of functional polymers that adsorb chlorophenols and their reactive metabolites such as DCHQ have the potential to not only segregate the toxic compounds from human tissues but also to kill breast cancer cells.

## Conclusions

Several cross-linked polymers that contain β-CD at variable composition of hexamethylene diisocyanate (HDI-X; X = 1, 3, and 6) were prepared and structurally characterized. The adsorption properties of the HDI-X polymers in aqueous solution were evaluated with pentachlorophenol (PCP) and other phenolic adsorbates [2,4,6-trichlorophenol (TCP); 2,4,6-trimethylphenol (TMP)] and compared with a commercial granular activated carbon (GAC) adsorbent. The HDI-X polymers and GAC adsorbents display variable uptake and selectivity toward the phenolic compounds, according to the sorption isotherms and % uptake profiles monitored via batch sorption studies and ESI-MS, along with further support by 1D/2D NMR results. While GAC reveals greater uptake of PCP due to complementary apolar interactions, the selectivity of HDI-3 surpassed that of GAC. The adsorption and *in-situ* biodegradation properties of a HDI-3 polymer-immobilized *Sphingobium Chlorophenolicum* (SpC) bacterium (HDI-3-SpC) with PCP and its reactive metabolites [6-dichloro-1,4-hydroquinone (DCHQ) and tetrachloro-1,4-benzoquinone (TCBQ)] were evaluated. The HDI-3-SPC system affords dual function; adsorption and *in-situ* biodegradation of DCHQ and TCBQ metabolites. A model compound (2,6-dichlorophenol; 2,6-DCP) was used as a proxy to support the uptake of DCHQ by the HDI-3 polymer. The apoptosis and cytotoxic effects of the PCP metabolites toward various cancer cell lines reveal that exposure to PCP and/or its metabolites may induce or inhibit cancer cell deaths, further illustrating the relevance of this dual function material for sustainable remediation of PCP and its metabolites. A key contribution of this study relates to the characterization of the adsorption properties of cross-linked polymer systems and their potential utility as bacterial supports for the *in-situ* removal and biodegradation of phenolic waterborne contaminants. The dual function properties of the polymer-bacterium system herein affords uptake and degradation of PCP, along with its oxidative metabolites such as DCHQ and TCBQ.

## Author contributions

LW conceived the study, supervised the research, and secured the research funding. AK carried out the primary structural, physicochemical studies, and contributed to the drafting of the manuscript. JY contributed to the toxicological assays and measurements related to bacterium biodegradation studies of chlorophenols. All co-authors contributed to editing of the manuscript.

### Conflict of interest statement

The authors declare that the research was conducted in the absence of any commercial or financial relationships that could be construed as a potential conflict of interest.

## Abbreviations

CD: Cyclodextrin
DCHQ: 6-dichloro-1,4-hydroquinone
DCP: Dichlorophenol
EDA: Electron-Donor Acceptor
HDI: Hexamethylene diisocyanate
PBS: Phosphate-Buffered Saline
PCP: Pentachlorophenol
ROESY: Rotating Frame Overhauser Spectroscopy
SpC: Sphingobium Chlorophenolicum
TCBQ: Tetrachloro-1,4-benzoquinone
TCP: Trichlorophenol
TMP: Trimethylphenol.
