# Supplementary material for: Cyclodextrin-Based Polymer-Supported Bacterium for the Adsorption and in-situ Biodegradation of Phenolic Compounds
Source: Front Chem. 2018 Sep 11;6:403. doi: 10.3389/fchem.2018.00403 (PMC6141685; doi:10.3389/fchem.2018.00403)
Supplement: Supplementary file 1 [file Table_1.DOCX]

Supplementary Material

Cyclodextrin-Based Polymer-Supported Bacterium for the Adsorption and *In-Situ* Biodegradation of Phenolic Compounds

Abdalla H. Karoyo, ^1^ Jian Yang, ^2^ & Lee D. Wilson^1^*

^1^Department of Chemistry, University of Saskatchewan, 110 Science Place, 156 Thorvaldson Building, Saskatoon, SK, S7N 5C9, Canada.

^2^Drug Discovery and Development Research Group, College of Pharmacy and Nutrition, University of Saskatchewan, 107 Wiggins Road, Saskatoon, S7N 5E5, Canada.

*** Correspondence:** Corresponding Author: [lee.wilson@usask.ca](mailto:lee.wilson@usask.ca)

# Table S1. Complexation-induced Chemical Shift changes of the adsorbent-adsorbate Systems

| **Chemical shifts (ppm) for the adsorbates/adsorbent signals** | | | | | | | | |
| --- | --- | --- | --- | --- | --- | --- | --- | --- |
|  | **H** | **CH_3_,(4)** | **CH_3_,(*2,6)*** | **H_3_** | **H_5_** | **H_α_** | **H_β_** | **H_γ_** |
| **TCP** | **6.66** | **-** | **-** | **-** | **-** | **-** |  |  |
| **TMP** | **6.81** | **2.10** | **2.09** | **-** | **-** | **-** |  |  |
| **HDI-1** | **-** | **-** | **-** | **3.86** | ****** | **3.05** | **1.43** | **1.27** |
| **HDI-1/TCP** | **7.20**  **(+0.54)** | **-** | **-** | **3.84**  **(-0.02)** | **3.71** | **3.06**  **(+0.01)** | **1.44**  **(+0.01)** | **1.28**  **(+0.01)** |
| **HDI-1/TMP** | **6.74**  **(-0.07)** | **2.09**  **(-0.01)** | **2.07**  **(-0.02)** | **3.84**  **(-0.02)** | **3.73** | **3.07**  **(+0.02)** | **1.45**  **(+0.02)** | **1.28**  **(+0.01)** |


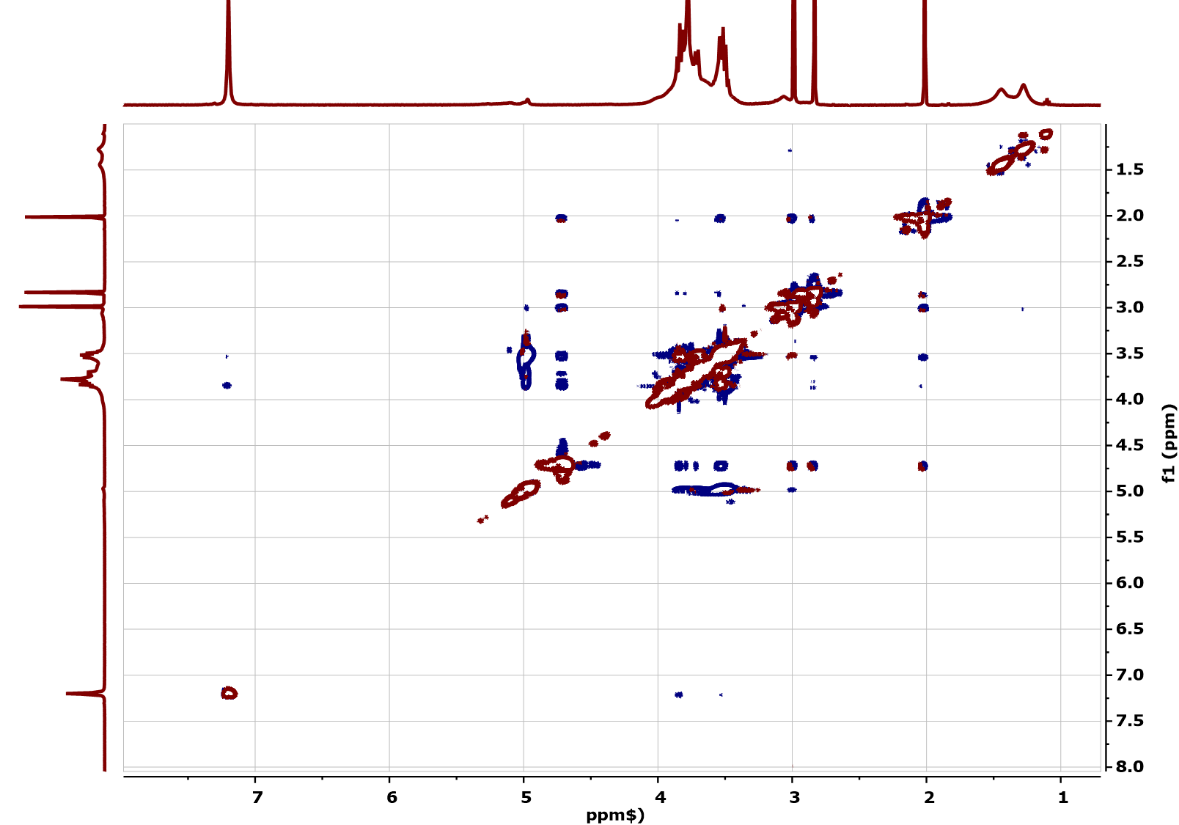

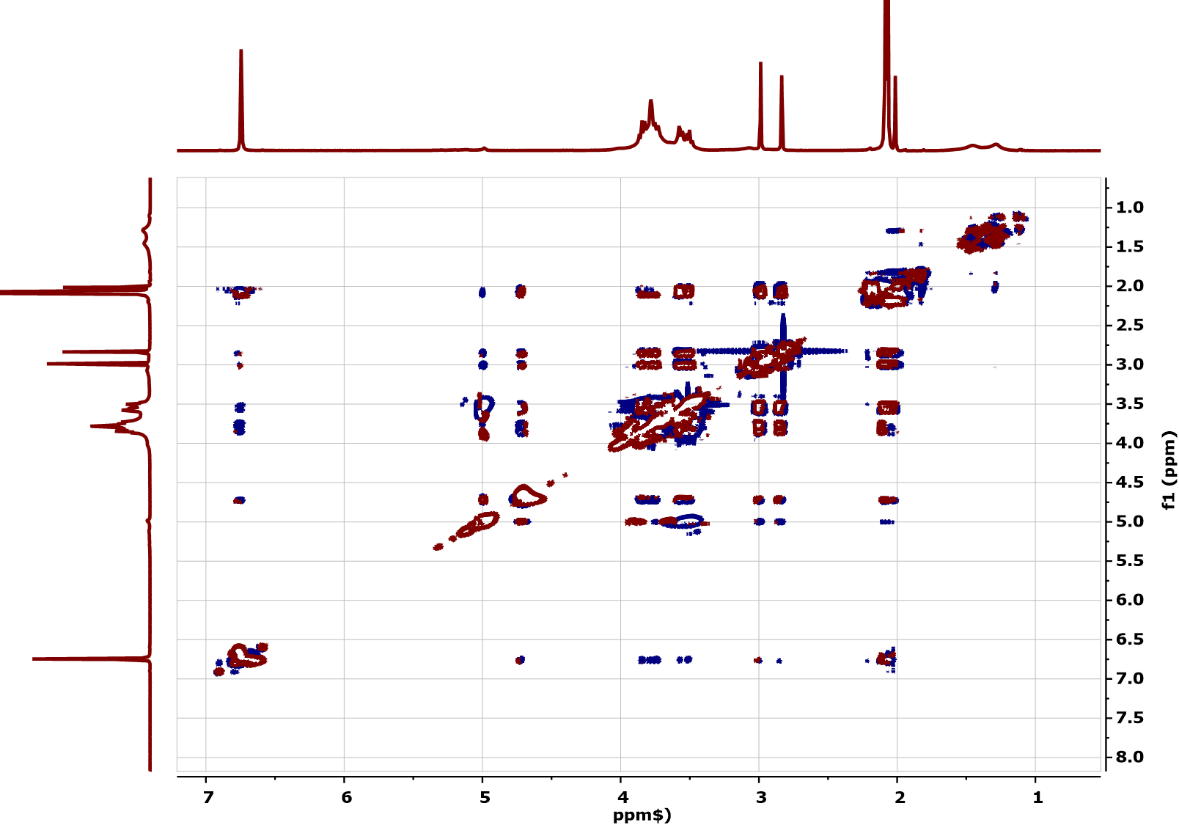


**(a)**

**(b)**

**Figure S1. 2D ROESY spectra for Cyclodextrin-based HDI-1 polymer adsorbent with (a) trichlorophenol (TCP) and (b) trimethylphenol (TMP).**
